# Characterization of pomegranate peel extracts obtained using different solvents and their effects on cell cycle and apoptosis in leukemia cells

**DOI:** 10.1002/fsn3.1831

**Published:** 2020-08-31

**Authors:** Leticia Tamborlin, Beatriz Rocchetti Sumere, Mariana Corrêa de Souza, Nathalie Fortes Pestana, Ana Carolina Aguiar, Marcos Nogueira Eberlin, Fernando Moreira Simabuco, Maurício Ariel Rostagno, Augusto Ducati Luchessi

**Affiliations:** ^1^ Laboratory of Biotechnology (BioTech) School of Applied Sciences (FCA) University of Campinas (UNICAMP) Limeira Brazil; ^2^ Institute of Biosciences (IB) São Paulo State University (UNESP) Rio Claro Brazil; ^3^ Multidisciplinary Laboratory of Food and Health (LabMAS) School of Applied Sciences (FCA) University of Campinas (UNICAMP) Limeira Brazil; ^4^ ThoMSon Mass Spectrometry Laboratory Institute of Chemistry (IQ) University of Campinas (UNICAMP) Campinas Brazil; ^5^ MackMass Laboratory School of Engineering (PPGEMN) Mackenzie Presbyterian University São Paulo Brazil

**Keywords:** apoptosis, biological activity, cell cycle, leukemia, pomegranate peel extracts, punicalagin

## Abstract

Pomegranate (*Punica granatum L*.) has been used in traditional herbal medicine by several cultures as an anti‐inflammatory, antioxidant, antihyperglycemic, and for treatment and prevention of cancer and other diseases. Different parts of the fruit, extraction methods, and solvents can define the chemical profile of the obtained extracts and their biological activities. This study aimed to characterize the chemical profile of peel extracts collected using different extraction solvents and their biological effects on the cell cycle and apoptosis of THP‐1 leukemic cells. Aqueous extract presented the highest content of punicalagins (*α* pun = 562.26 ± 47.14 mg/L and *β* pun = 1,251.13 ± 22.21 mg/L) and the lowest content of ellagic acid (66.38 ± 0.21 mg/L), and it promoted a significant impairment of the cell cycle S phase. In fact, punicalagin‐enriched fraction, but not an ellagic acid‐enriched fraction, caused an S phase cell cycle arrest. All extracts increased the number of apoptotic cells. Punicalagin‐enriched fraction increased the percentage of cells with fragmented DNA, which was intensified by ellagic acid combination. The treatment combining punicalagin and ellagic acid fractions increased the apoptotic cleaved PARP1 protein and reduced the activation of the growth‐related mTOR pathway. Thus, these results evidence that solvent choice is critical for the phenolic compounds profile of pomegranate peel extracts and their biological activities.

## Graphical abstract

The main aim of this study was to assess and compare the differences in the chemical profile of pomegranate peel extracts obtained using different extraction solvents and how does it affect their biological properties.

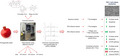

## 1 INTRODUCTION

Pomegranate *(Punica granatum L.)* is a native plant from the Middle East, which has been used in traditional herbal medicine by several cultures (Adhami, Khan, & Mukhtar, 2009; Jurenka, 2008). The plant is divided into seven anatomical parts: seeds, juice (arils), peels, leaves, flowers, barks, and roots. Each part has been found to display unique and attractive pharmacological activities (Lansky & Newman, 2007). Pomegranate therapeutic properties are wide‐ranging, including anti‐inflammatory, antioxidative, antihyperglycemic, and antihyperlipidemic effects. Due to it, pomegranate juice or extracts have been used as a complement for the treatment and prevention of cancer, cardiovascular diseases, and others (El‐Hadary & Ramadan, 2019; Jurenka, 2008; Karwasra et al., 2019; Lansky & Newman, 2007; Orgil, Spector, Holland, Mahajna, & Amir, 2016). Pomegranate has shown potential in the treatment of several tumors by inhibition of proliferation in colon, breast, lung, pancreatic, and prostate cancer cells* in vitro* (Adhami et al., 2009; Panth, Manandhar, & Paudel, 2017). However, few studies have investigated the potential of pomegranate, let alone its peel extracts, for the treatment or prevention of leukemia (Asmaa, Ali, Farid, & Azman, 2015; Dahlawi, Jordan‐Mahy, Clench, & Le Maitre, 2012; Dahlawi, Jordan‐Mahy, Clench, McDougall, & Maitre, 2013).

**FIGURE 1:**
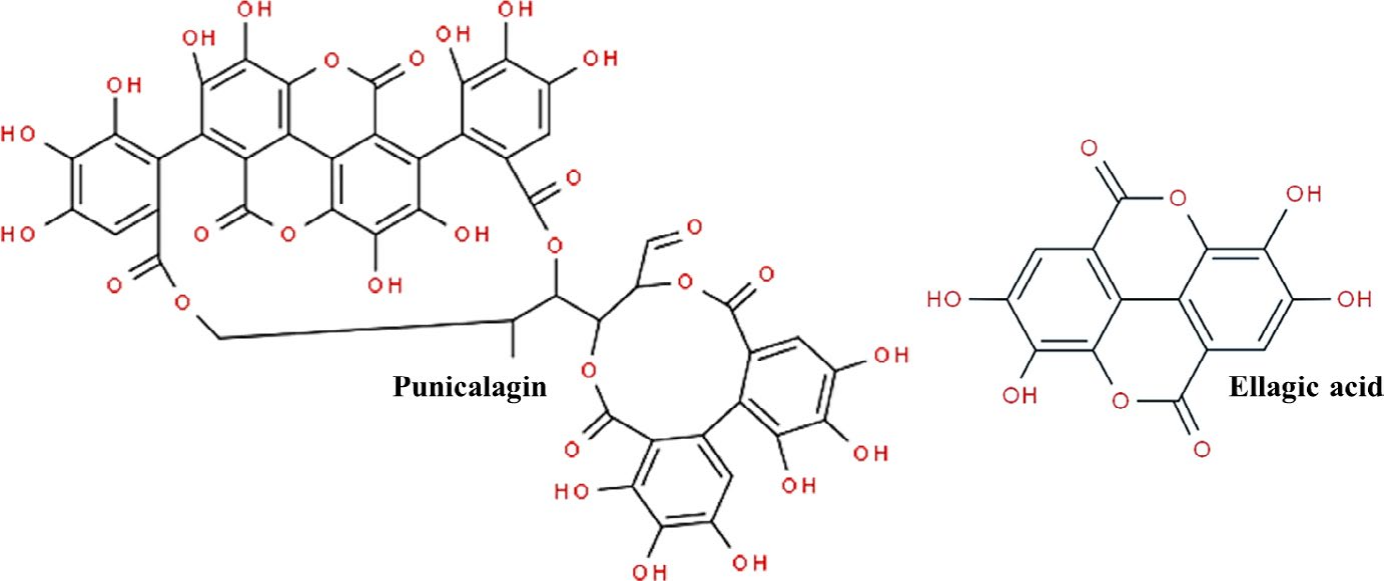
Chemical structure of the main phenolic compounds of pomegranate peel obtained from “ChemSpider: The Free Chemical Database” ("ChemSpider: The Free Chemical Database," 2012). Punicalagin and ellagic acid are the main compounds present in the pomegranate peel

Most of the scientific reports regarding pomegranate and cancer have focused on the edible parts of the fruit (seeds and juice). Recently, it has been given more attention to its nonedible parts, such as peels, leaves, flowers, barks, and roots (Akhtar, Ismail, Fraternale, & Sestili, 2015; Asmaa et al., 2015; El‐Hadary & Ramadan, 2019; Fischer, Carle, & Kammerer, 2011; Li et al., 2016; Song, Li, & Li, 2016). The pomegranate peel is considered byproduct for food and beverage industries even though it has been shown to display greater antioxidant activity than the edible parts of the fruit (Fischer et al., 2011; Yunfeng Li et al., 2006). The peel contains the most promising pool of phenolic compounds when compared to other parts of the fruit (Akhtar et al., 2015). It is the main source of bioactive compounds, such as flavonoids, ellagitannins, and proanthocyanidins. Ellagitannins are the predominant phenolic class, in which punicalagins and ellagic acid (Figure 1) are the main present compounds (Akhtar et al., 2015; Fischer et al., 2011; Khalil, Khan, Shabbir, & Khalil, 2018).

These bioactive compounds need to be extracted from the raw material matrix to have pharmacological applications. Different types of solvents and techniques are available for extraction. The choice of solvent should be considered according to the particular characteristics of the sample and target compounds (M. Rostagno, D’Arrigo, & Martínez, 2010; M. Rostagno, Villares, Guillamón, García‐Lafuente, & Martinez, 2009). Due to the polarity of phenolic compounds from pomegranate peels, the most used solvents are water or its hydroalcoholic mixtures (Singh et al., 2014; Venkataramanamma, Aruna, & Singh, 2016). The solvent used for the extraction is determinant to the final chemical profile of the extract. Consequently, the relative and absolute concentrations of the extracted compounds will also significantly affect their bioactivity (Rostagno, Prado, & Kraus, 2013).

Considering the importance of the extraction solvent for the extract chemical profile, and its influence on their biological activity, this study aimed to characterize and compare the chemical profile and biological activity of extracts obtained using different extraction solvents. The extracts were tested against THP‐1 leukemic cells, and it was also determined the relationship with their chemical profile.

## 2 MATERIALS AND METHODS

### 2.1 Processing of the pomegranate peel

The extracts used in this study were obtained from the peels of pomegranate fruits (Wonderful variety) purchased at a local store in Limeira‐SP (Brazil). Peels were separated from the rest of the fruit using a depulper (Des‐60 Braesi, Caxias do Sul, RS, Brazil). Then, they were dried at 50°C for 48 hr on a laboratory oven. The dried peels were ground and sieved before being stored in amber glass vials at −20°C until used as raw material.

### 2.2 Preparation of the pomegranate peel extracts

The preparation of the extracts using different solvents was carried out by ultrasound‐assisted pressurized liquid extraction (UAPLE) on a multipurpose analysis system (Extract‐US system—FAPESP 2013/043044—patent pending) described in a previous study (Sumere et al., 2018). The system was configured to “extraction” mode by changing the position of the automatic valves. The extractions were carried out using 1.0 g of sample and different solvents: water (solvent A), ethanol 30% in water v/v (solvent B), ethanol 50% in water v/v (solvent C), and ethanol 70% in water v/v (solvent D). All extractions were carried out in static mode for 30 min at 70°C and 100 bar with the assistance of ultrasound (generator set at 400 W). About 17 ml of extracts was obtained in each extraction. The extracts were filtered through a 0.20 μm nylon syringe filter (Analitica, São Paulo, SP, Brazil) and stored at −20°C before being used in the experiments and chromatographic analysis.

### 2.3 On‐line separation of the phenolic compounds

The separation of compounds from the sample in different fractions was also carried out in the Extract‐US system by high‐performance liquid chromatography (HPLC). For this, an HPLC column (4.6 × 50 mm) packed with a solid‐phase adsorbent (Sepra C18‐E, Silica Base, 50 μm) was coupled on‐line after the extraction cell and a two‐stage process was performed. The system was initially configured to “solid‐phase extraction” mode for the activation of the adsorbent with 30 ml of methanol and conditioning with 30 ml of water. Afterward, the system was configured to “Extraction” mode, and 1.5 g of sample was extracted in dynamic mode at 40°C using 150 ml of water as the solvent. In this stage, polar compounds, such as *α* punicalagin (*α* pun) and *β* punicalagin (*β* pun), poorly interacted with the adsorbent. Then, they were extracted and collected in the aqueous fraction. Moderately polar compounds, such as ellagic acid (EA) and its derivatives, were retained by the adsorbent. After the first stage, the second stage begun by changing the solvent composition to ethanol, which extracted the remaining moderately polar compounds from the sample. It simultaneously eluted the retained compounds by the adsorbent, producing a concentrated extract rich in ellagic acid and derivatives. After the extraction, extracts were concentrated by rotatory evaporation and dissolved in ethanol 50%. The extracts were filtered through a 0.20 μm nylon syringe filter (Analitica, São Paulo, SP, Brazil) and stored at −20°C before being used in the experiments and chromatographic analysis.

### 2.4 Identification of phenolic compounds in the extracts

The identity of the compounds found in the extracts was determined by ultra‐high‐performance liquid chromatography‐tandem mass spectrometer (UHPLC‐MS/MS) on an 8,040 Shimadzu system (Kyoto, Japan) equipped with a triple quadrupole mass spectrometer with an electrospray ionization (ESI) source. The separation of the compounds found in the sample was carried out on a C_18_ Kinetex 2.6 μm, 3.0 mm i.d., 100 mm column (Phenomenex, Torrance, CA, USA) based on the conditions of a previously developed method (M. Rostagno et al., 2011). The separation was achieved with a flow rate of 0.3 ml/min and the following gradient of water (solvent A ‐ 0.1% formic acid) to acetonitrile (solvent B‐ 0.1% formic acid): 0 min 100% A; 2 min 90% A; 7 min 85% A; 13 min 70% A; 16 min 70% A; 20 min 60% A; and 24 min 20% A. The chromatograms were monitored between 240 and 400 nm, and the areas were registered at 260 nm. The volume of the sample was 10 μl. The capillary voltage was set to −3.5 kV, while heat block temperature was set to 500°C; desolvation line temperature was 250°C; drying gas flow (N_2_) was 10 L/min; nebulizing gas flow (N_2_) was 1.5 L/min; and collision‐induced dissociation gas pressure (Ar) was 224 kPa. Initially, ESI(‐)‐MS/MS data were collected for the deprotonated molecule [M − H]−, and two of its most selective product ions were chosen for the MRM transitions using 20 ms of dwell time. It was used the LabSolutions software (version 5.53 SP2, Shimadzu). The identification of compounds was based on their mass (*m/z*), retention time, and co‐elution with the available authentic standards.

### 2.5 Quantitation of phenolic compounds in the extracts

The quantitation of the compounds found in the extracts was also carried out in the Extract‐US system by HPLC. For this, the system was set to “Analysis” mode. Water (solvent A) and methanol (solvent B), both with 0.1% of phosphoric acid, were used as mobile phases. The separation was achieved at 0.9 ml/min on a Kinetex C18 column (2.6 μm, 100 × 4.6 mm, Phenomenex) using the following gradient: 8.15 min, 90% A; 12.6 min, 70% A; 21.6 min, 50% A; 25 min, 10% A; 29 min, 10% A and 30 min, 96% A. The volume of the sample was 5 μl. Peak areas were recorded at 378 nm. The identity of each compound was confirmed by comparison of the retention time with authentic standards. Six‐point calibration curves were constructed with concentration ranging from approximately 5 mg/L and 110 mg/L. Results were expressed as a concentration in mg/L.

### 2.6 Cell culture

The human monocyte cell line (THP‐1), derived from a patient with acute monocytic leukemia, was acquired from the laboratory of Prof. Dr. Rui Curi (ICB—USP, São Paulo, SP, Brazil). The cells were grown in Roswell Park Memorial Institute (RPMI)‐1640 medium supplemented with 10% fetal bovine serum (FBS), L‐glutamine (2 mM), penicillin (100 U/ml), and streptomycin (100 µg/ml) (Thermo Fisher Scientific, Carlsbad, CA, USA) in a humidified atmosphere at 37°C containing 5% CO_2_.

### 2.7 Cell treatment

THP‐1 cells were seeded at a density of 3ₓ10^5^ cells per well in 24‐well plates and grown for 24 hr in the regular culture medium before the treatment. The cells were treated with a fixed volume of 18 µl of each extract (A, B, C, and D) added to 1 ml of regular culture medium for 48 hr. The volume was defined and fixed to avoid not exceed 1.25% ethanol final concentration in the well. All extracts were diluted with their proper solvents as following. For example, it was used 9 μl of concentrated A extract (aqueous extract) plus 9 μl of water to adjust the final volume of treatment to 18 μl, and so forth for the other extracts. The solvents were also used to treat the control samples. Moreover, it was also performed treatments with the isolated compounds from pomegranate peel extracts, such as ellagic acid (EA), punicalagin (Pun), and ellagic acid + punicalagin (EA + Pun) fractions. The cells were treated with a fixed volume of 18 µl of each extract (EA, Pun, and EA + Pun) added to 1 ml of regular culture medium for 48 hr. Fixed volume was defined to establish the final ethanol concentration at 0.9% in each well. The samples were diluted to adjust the volume as follows: it was used 9 μl of EA extract (in ethanol 70%) plus 9 μl of ethanol 30%; 9 μl of Pun extract (in ethanol 30%) plus 9 μl of ethanol 70%; and 9 μl of EA extract (in ethanol 70%) plus 9 μl of Pun extract (in ethanol 30%) for the EA + Pun treatment. It was used 9 μl of ethanol 70% plus 9 μl of ethanol 30%, to treat the control samples. After the treatments, the cell suspensions were collected for the analysis by flow cytometry and immunoblotting.

### 2.8 Cell cycle and DNA fragmentation analysis by flow cytometry

The cell cycle distribution and percentage of cells with fragmented DNA were evaluated by the DNA content assessment using flow cytometry, following the protocol of propidium iodide (PI) labeling. After the treatment period, the cells were harvested, washed with phosphate‐buffered saline (PBS), and fixed with cold ethanol 70% in the ice bath for 30 min. Then, they were stained with propidium iodide (20 μg/ml) diluted in PBS containing RNase A (10 μg/ml) and Triton X‐100 (0.1%) at room temperature for 30 min in the dark. The stained cells were analyzed in the BD Accuri ™ C6 flow cytometer (BD Biosciences, San Jose, CA, USA) using the FL‐2A channel to measure the cell DNA content. It was recorded 10.000 events per sample. Cell percentages in the different cell cycle phases were determined from the collected data. Each experiment was performed in a sample triplicate. Three independent experiments (biological replicates) were performed to carry out the statistical analyses.

### 2.9 Immunoblotting

THP‐1 cells were collected from the plates and disrupted using lysis buffer (100 mM Tris/HCl pH 7.4; 10 mM Na_2_ ethylenediaminetetraacetic acid [EDTA] pH 8.0; 1 mM DL‐Dithiothreitol [DTT]; 1% Triton X‐100; and 1x complete EDTA‐free protease inhibitor cocktail [Roche Diagnostics GmbH, Mannheim, Germany]). About 30 µg of total protein extract was resolved by 12% sodium dodecyl sulfate–polyacrylamide gel electrophoresis (SDS–PAGE). The proteins were transferred to nitrocellulose membranes and blocked with tris‐buffered saline with Tween^®^ 20 (TBS‐T) containing 5% nonfat milk. The membranes were incubated overnight at 4°C with the desired primary antibodies diluted in TBS‐T containing 5% bovine serum albumin (BSA). The next day, the membranes were washed with TBS‐T and incubated with the specific anti‐immunoglobulin G (IgG) horseradish peroxidase‐conjugated antibody diluted in TBS‐T containing 5% nonfat milk for 1 hr at room temperature. Then, they were rinsed again with TBS‐T before the detection of the immunoreactive proteins. The equipment G: BOX Chemi XRQ photodocumentator powered by GeneSys software (SYNGENE, Frederick, MD, USA) was used to detect the chemiluminescence signal.

### 2.10 Statistical analyses

The obtained results are from three independent experiments with cells from different batches. Compounds concentration data are presented as mean ± standard deviation (*SD*). Flow cytometry and immunoblotting data are presented as mean ± standard error of the mean (*SEM*). Different treatments were compared by one‐way analysis of variance (ANOVA) followed by Tukey post hoc test (parametric data). Statistical analyses were carried out using SigmaStat software version 3.5 for Windows (Systat Software, Inc., Point Richmond, CA, USA). Statistically significant differences were considered for p‐values less or equal than 0.05 (*p* < .05). The symbol * in the figures and the upper case letters (^A^, ^B^, ^C^, and ^D^) in the table indicate statistically significant differences between the means. The graphs were carried out using GraphPad Prism 7.00 for Windows (GraphPad Software, La Jolla, CA, USA).

## 3 RESULTS

### 3.1 The chemical profile of the obtained extracts was significantly influenced by the use of different solvents

**FIGURE 2:**
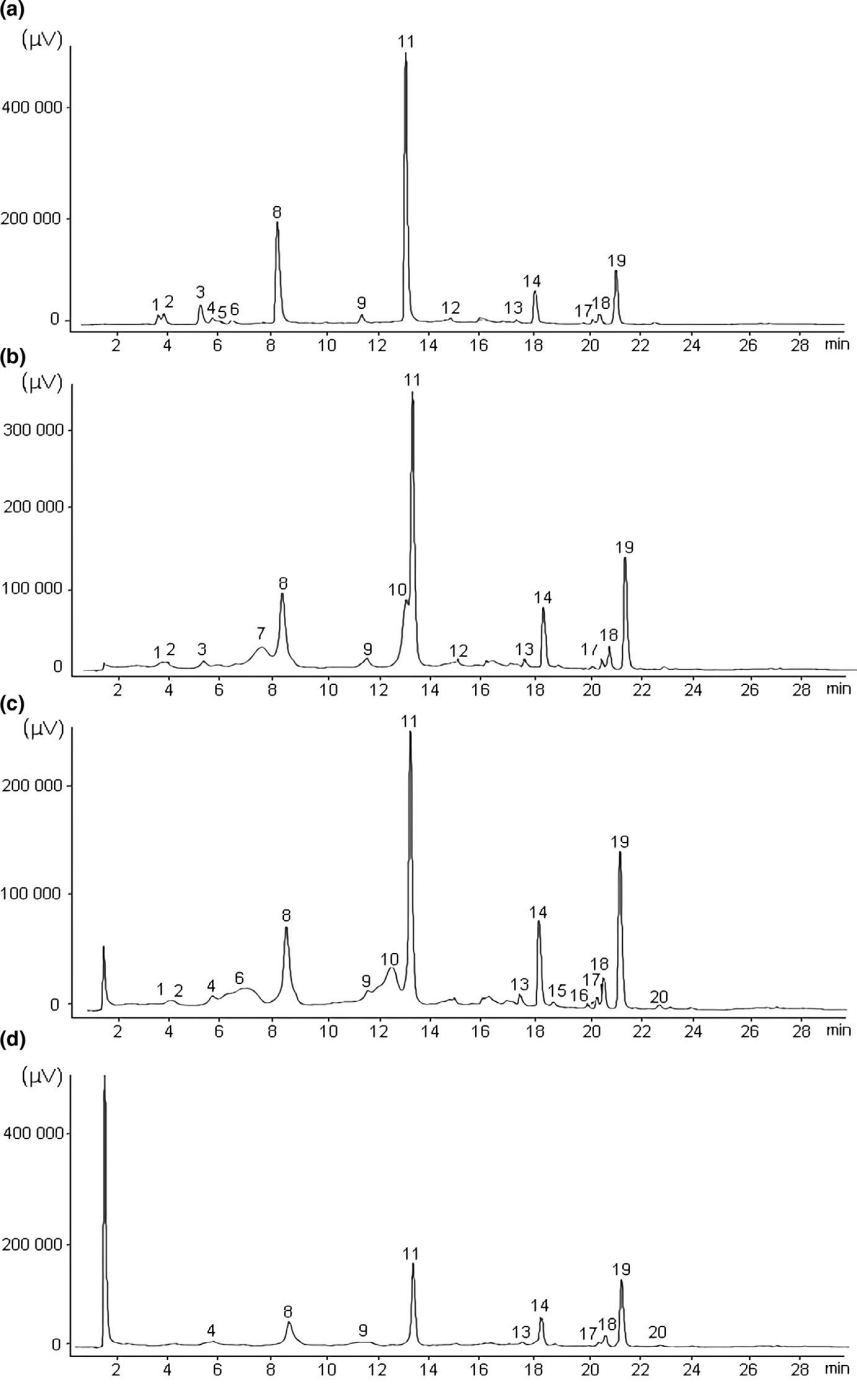
Representative chromatograms in 370 nm of the HPLC analysis from pomegranate peel extracts obtained by using different extraction solvents. The extractions were performed using four different solvents: (a) water, (b) 30% ethanol, (c) 50% ethanol, and (d) 70% ethanol

Compounds of pomegranate peel were extracted using different solvents for the extraction: (A) water, (B) 30% ethanol in water v/v, (C) 50% ethanol in water v/v, and (D) 70% ethanol in water v/v. Figure 2 shows the HPLC chromatograms of the obtained extracts recorded at 370 nm.

The use of different solvents for the extraction of the compounds from pomegranate peel resulted in relatively similar chromatograms, with approximately 20 compounds in all extracts (Figure 2). Four main compounds were identified in all extracts (Table 1): *α* punicalagin (*α* pun, MW 1,083, retention time 8.3 min, peak 8), *β* punicalagin (*β* pun, MW 1,083, retention time 13.1 min, peak 11), ellagic acid hexoside (EA‐hex, MW 463, retention time 18.0 min, peak 14), and ellagic acid (EA, MW 301, retention time 21.1 min, peak 19). Other identified compounds include ellagic acid pentoside (MW 443, retention time 20.2 min, peak 17), ellagic acid deoxyhexoside (MW 447, retention time 20.5 min, peak 18), and pedunculagin I (MW 783, retention time 22.6 min, peak 20).

**TABLE 1:** Chemical characterization by UHPLC‐MS/MS of the most representative compounds from pomegranate peel extracts

	Compound number	Assignment	Retention time (min)	UHPLC–ESI(–) MS^2^ experiment	[M ‐ H]^‐^ *m/z*
I	8	*α* punicalagin	8.3	301, 575, 601, 781	1,083
II	11	*β* punicalagin	13.1	300, 601, 781	1,083
III	14	Ellagic acid‐hex	18.0	300, 301, 302	463
IV	17	Ellagic acid‐pent	20.2	300, 301	443
V	18	Ellagic acid‐deoxyhex	20.5	300, 301, 302	447
VI	19	Ellagic acid	21.1	174, 185, 229, 301	301
VII	20	Pedunculagin I	22.6	301, 481	783

Some compounds were not present in all extracts, and it was observed significant differences in the concentration of those that were identified. For example, peaks 1 and 2 were not present in the extract obtained using the solvent D, and the peak 20 was not present in the extracts using the solvents A and B. Peaks 7, 10, 15, 16, and 20 were not detected in the A extract, whereas peaks 4, 5, 6, 15, 16, and 20 were not detected in the B extract. Peaks 3, 5, 7, and 12 were not detected in the C extract whereas peaks 1, 2, 3, 5, 6, 7, 10, 12, 15, and 16 were not detected in the D extract.

By far the major difference between extracts was the relative concentrations of the extracted compounds (Table 2). Compounds found in higher concentrations were *α* pun, *β* pun, ellagic acid‐hex, and ellagic acid. The highest concentration of *α* pun and *β* pun was observed in the A extract and the lowest in the D extract. Curiously, the highest concentration of ellagic acid‐hex was found in the B extract and the lowest concentration in the D extract.

**TABLE 2:** Concentration (mg/L) of the compounds detected by HPLC in extracts obtained from pomegranate peel by using different solvents

		Solvents		
Compound	Water	EtOH 30%	EtOH 50%	EtOH 70%
1	42.62 ± 2.30^A^	31.74 ± 2.37^B^	9.58 ± 0.81^C^	‐
2	58.21 ± 1.98^A^	30.09 ± 1.50^B^	14.60 ± 2.31^C^	‐
3	113.02 ± 2.34^A^	29.14 ± 0.12^B^	‐	‐
4	35.62 ± 0.44^B^	‐	16.70 ± 0.88^C^	62.04 ± 6.46^A^
5	25.38 ± 0.84	‐	‐	‐
6	30.77 ± 0.18^B^	‐	202.66 ± 11.92^A^	‐
7	‐	296.22 ± 31.76	‐	‐
8 (*α* pun)	562.26 ± 47.14^A^	477.37 ± 26.41^B^	318.67 ± 31.90^C^	259.09 ± 19.71^C^
9	41.41 ± 2.21^B^	49.28 ± 0.97^B^	8.71 ± 0.49^C^	123.37 ± 5.73^A^
10	‐	388.22 ± 10.58^A^	201.60 ± 15.15^B^	‐
11 (*β* pun)	1,251.13 ± 22.21^A^	1,112.63 ± 54.89^B^	634.87 ± 37.36^C^	506.55 ± 4.68^D^
12	0.58 ± 0.10^B^	0.90 ± 0.01^A^	‐	‐
13	1.88 ± 0.08^B^	3.69 ± 0.17^A^	3.58 ± 0.10^A^	1.84 ± 0.11^B^
14 (Ellagic acid‐hex)	40.25 ± 0.20^B^	49.68 ± 0.18^A^	48.49 ± 0.36^A^	36.25 ± 1.57^C^
15	‐	‐	1.01 ± 0.01	‐
16	‐	‐	0.19 ± 0.01	‐
17	2.52 ± 0.01^C^	3.49 ± 0.00^A^	3.36 ± 0.04^A^	3.14 ± 0.12^B^
18	11.91 ± 0.19^B^	16.04 ± 0.10^A^	15.28 ± 0.32^A^	12.77 ± 1.17^B^
19 (Ellagic acid)	66.38 ± 0.21^C^	93.01 ± 0.61^A^	91.10 ± 0.20^AB^	88.87 ± 2.90^B^
20	‐	‐	1.38 ± 0.01^A^	1.21 ± 0.09^B^
**Σ** *	**1,843.23 ± 16.06^A^**	**1,732.69 ± 27.69^B^**	**1,093.13 ± 69.81^C^**	**890.76 ± 25.18^D^**
**Ʃt** **	**2,299.98 ± 19.71^B^**	**2,581.50 ± 9.95^A^**	**1,571.79 ± 70.80^C^**	**1,095.11 ± 25.94^D^**

* Σ sum of the four main compounds (*α* pun, *β* pun, Ellagic acid‐hex, and Ellagic acid).

** Ʃt sum of all peaks of phenolic compounds.

*** Results expressed by the mean ± standard deviation (*SD*).

**** The upper case letters refer to the variation of the solvent for each compound. Statistically significant differences have 5% level of significance by the Tukey test (*p* < .05).

When considering the total concentration of these four main phenolic compounds in the extracts obtained with the different solvents, A extract (water solvent) provided the highest extraction yield (Ʃ 1843.23 ± 16.06 mg/L). In contrast, when considering all detected phenolic compounds, B extract (30% ethanol solvent) was able to extract the highest amount of total phenolic compounds (Ʃt 2,581.50 ± 9.95 mg/L) as shown in Table 2.

### 3.2 Differences in the chemical profile of the obtained extracts lead to distinct effects on the S phase cell cycle arrest in leukemic cells

**FIGURE 3:**
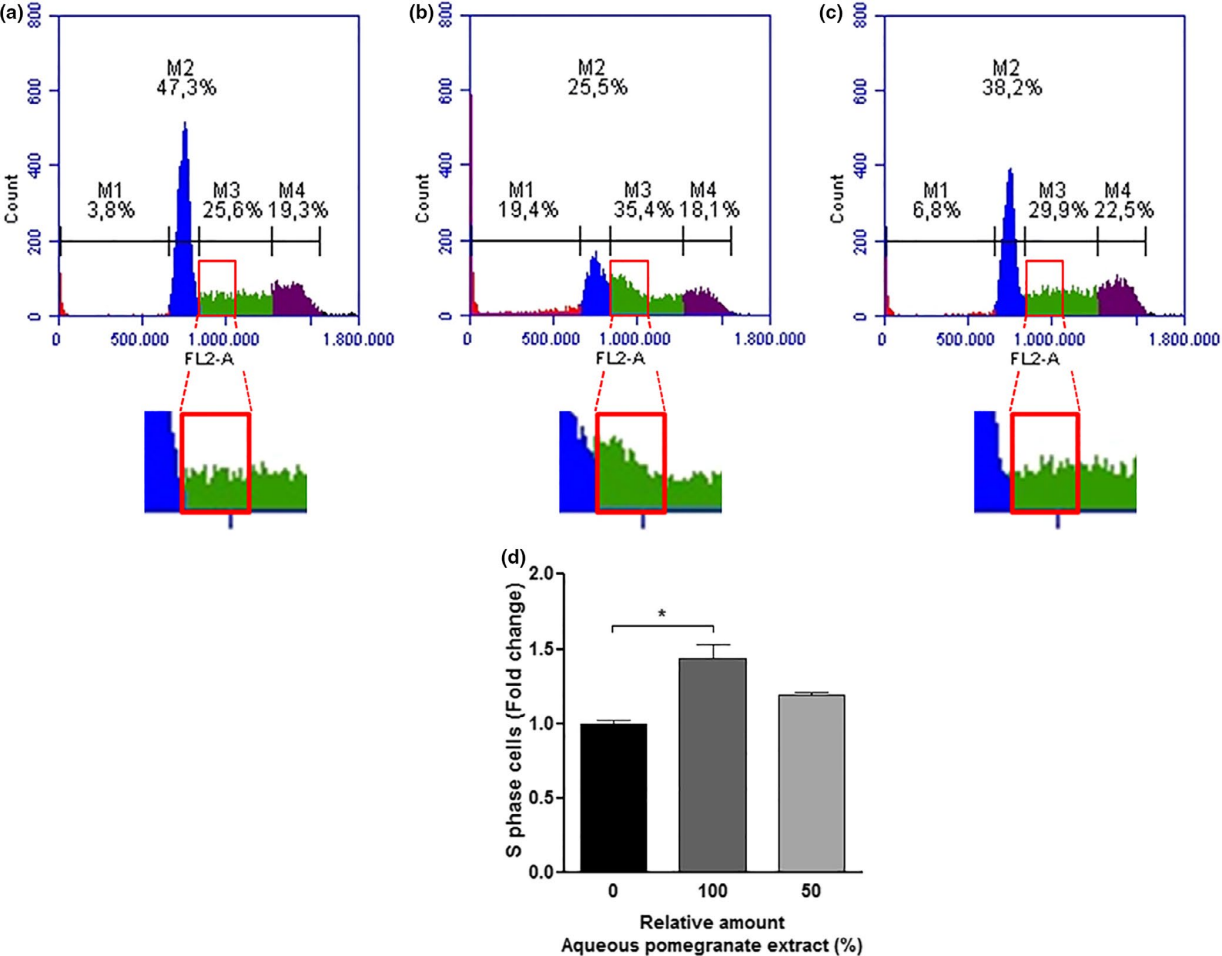
Cell cycle progression of THP‐1 cells treated with pomegranate peel extract A (aqueous extract) for 48 hr. (a) Control sample: THP‐1 cells were treated with 18 µl of water (vehicle). (b) Treated sample: THP‐1 cells were treated with 18 µl of extract A. (c) Treated sample: THP‐1 cells were treated with 18 µl of a diluted extract A (9 µl of extract + 9 µl of water). In all samples, the cells were treated for 48 hr, and the cell cycle distribution was evaluated after propidium iodide (PI) staining. Data are representative of three independent experiments. (d) Analysis of the percentage of cells in S phase. The results were presented in the fold change as mean ± *SEM* of three independent experiments. *indicates significant differences (*p* < .05)

**FIGURE 4:**
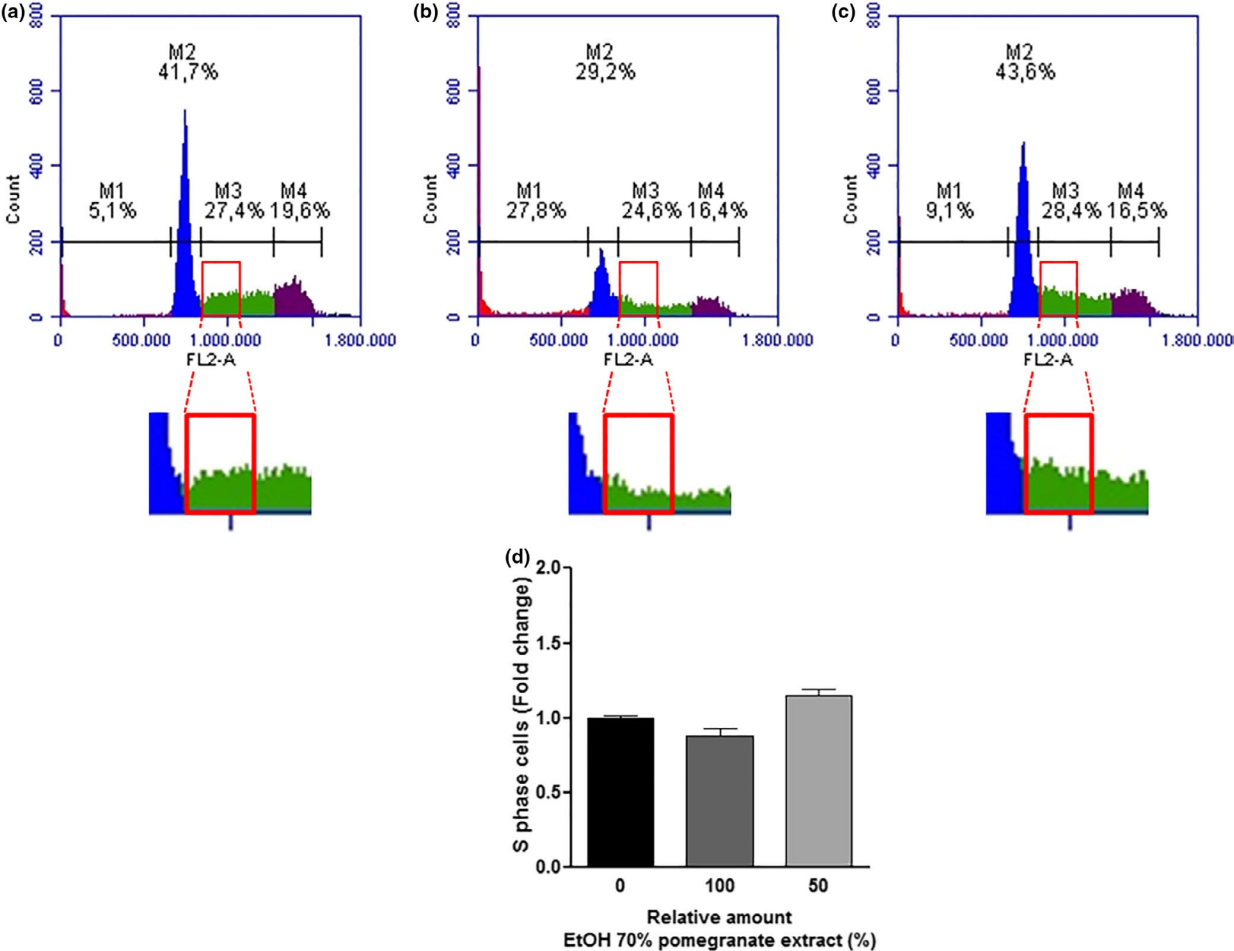
Cell cycle progression of THP‐1 cells treated with pomegranate peel extract D (70% ethanol extract) for 48 hr. (a) Control sample: THP‐1 cells were treated with 18 µl of 70% ethanol (vehicle). (b) Treated sample: THP‐1 cells were treated with 18 µl of extract D. (c) Treated sample: THP‐1 cells were treated with 18 µl of a diluted extract D (9 µl of extract + 9 µl of 70% ethanol). In all samples, the cells were treated for 48 hr, and the cell cycle distribution was evaluated after propidium iodide (PI) staining. Data are representative of three independent experiments. (d) Analysis of the percentage of cells in S phase. The results were presented in the fold change as mean ± *SEM* of three independent experiments. * indicates significant differences (*p* < .05)

THP‐1 human monocytic leukemia cells were treated with the obtained extracts for 48 hr, to verify whether these extracts had biological effects on the cell cycle progression. Afterward, the cells were analyzed by flow cytometry using the protocol for DNA labeling with propidium iodide. We compared the effects of the treatment using the extracts A (most polar solvent) and D (nonpolar solvent). Pomegranate peel A extract was able to induce a cell cycle arrest in the phase that controls DNA duplication (S phase), while the D extract was not (Figures 3 and 4). This finding seems to be dependent on the amount of the main phenolic compounds found in these extracts (*α* pun, *β* pun, ellagic acid‐hex, and ellagic acid), specifically of punicalagins. The punicalagins are present in higher concentrations in the extract produced using water as solvent (A extract) than the extract produced using 70% ethanol (D extract) (Table 2). The treatment with A extract significantly impaired the cell cycle progression, increasing the percentage of cells in the S phase by 44% (Figure 3). However, no statistically significant differences were observed in the cell cycle progression in the treatment with D extract (Figure 4).

### 3.3 Pomegranate peel extracts increased the percentage of cells with fragmented DNA

**FIGURE 5:**
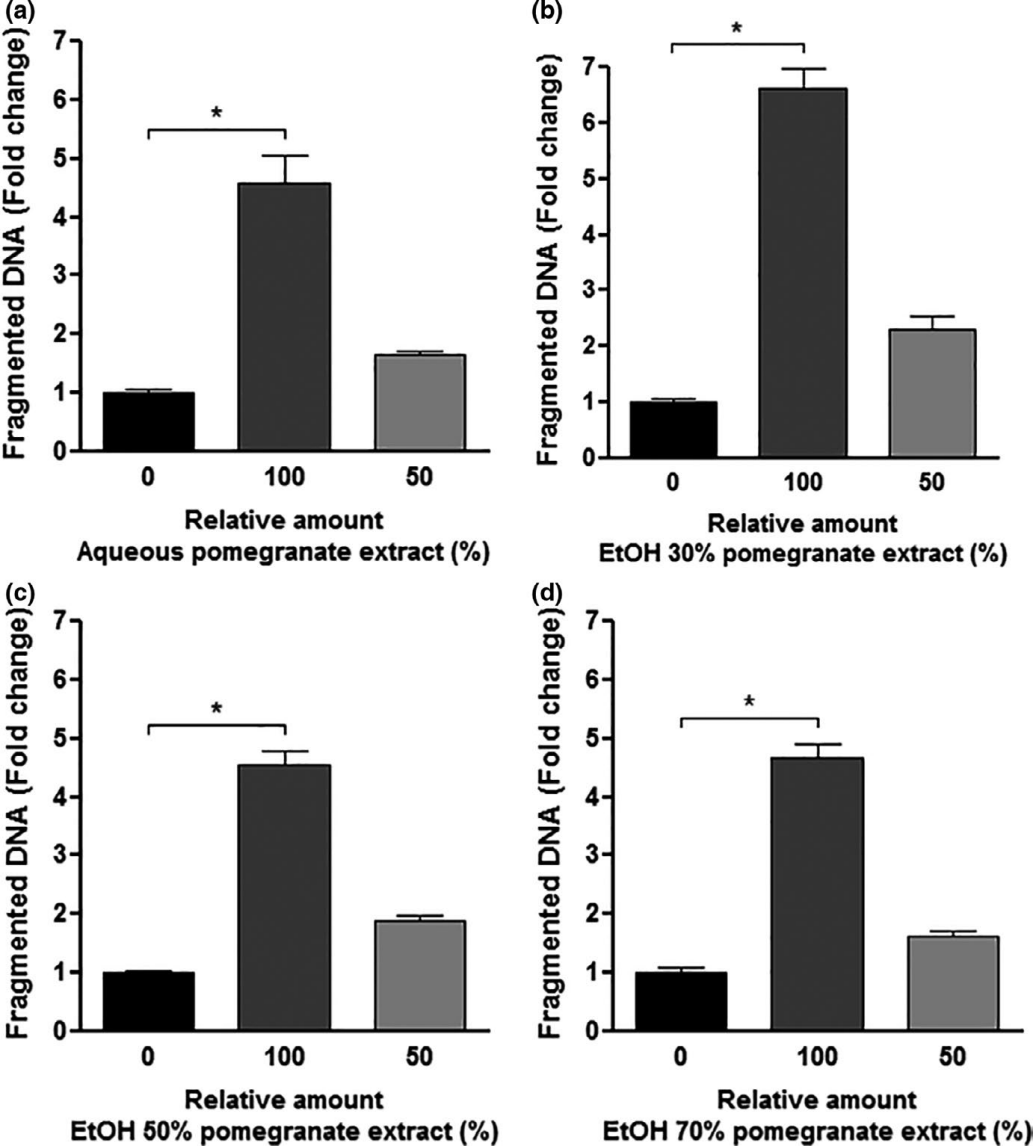
Percentage of cells with fragmented DNA in THP‐1 cells treated with all obtained extracts for 48 hr. In all frames, the black bars are the control sample treated just with the vehicles (solvents), dark gray bars are the cells treated with 18 µl of extracts, and light gray bars are the cells treated with 18 µl of a diluted extract (9 µl of extract + 9 µl of the vehicles). (a) Aqueous pomegranate extract. (b) 30% ethanol pomegranate extract. (c) 50% ethanol pomegranate extract. (d) 70% ethanol pomegranate extract. In all samples, the cells were treated for 48 hr, and the cell cycle distribution was evaluated after propidium iodide (PI) staining. The results were presented in the fold change as mean ± *SEM* of three independent experiments. *indicates significant differences (*p* < .05)

In addition to the S phase impairment, we observed a significant increase in the number of cells with fragmented DNA when they were treated with all extracts for 48 hr (Figure 5). The highest raise was observed in the treatment with B extract (30% ethanol). On these samples, the percentage of cells with fragmented DNA increased about 6.5 times in comparison to control samples, while in the other treatments, it was almost five times. The main difference among the extracts was the concentration of total phenolic compounds, in which the B extract was the one displaying the highest sum (Ʃt 2,581.50 ± 9.95 mg/L) (Table 2). The B extract also presented the highest amount of ellagic acid (93.01 ± 0.61 mg/L) and ellagic acid‐hex (49.68 ± 0.18 mg/L) (Table 2). These findings suggest that the highest concentration of total phenolic compounds, mainly of ellagic acid and its derivatives, was responsible for the increase in the percentage of cells with fragmented DNA.

### 3.4 Phenolic compounds separation reveals distinct effects for the main compounds present in the pomegranate peel extracts

**FIGURE 6:**
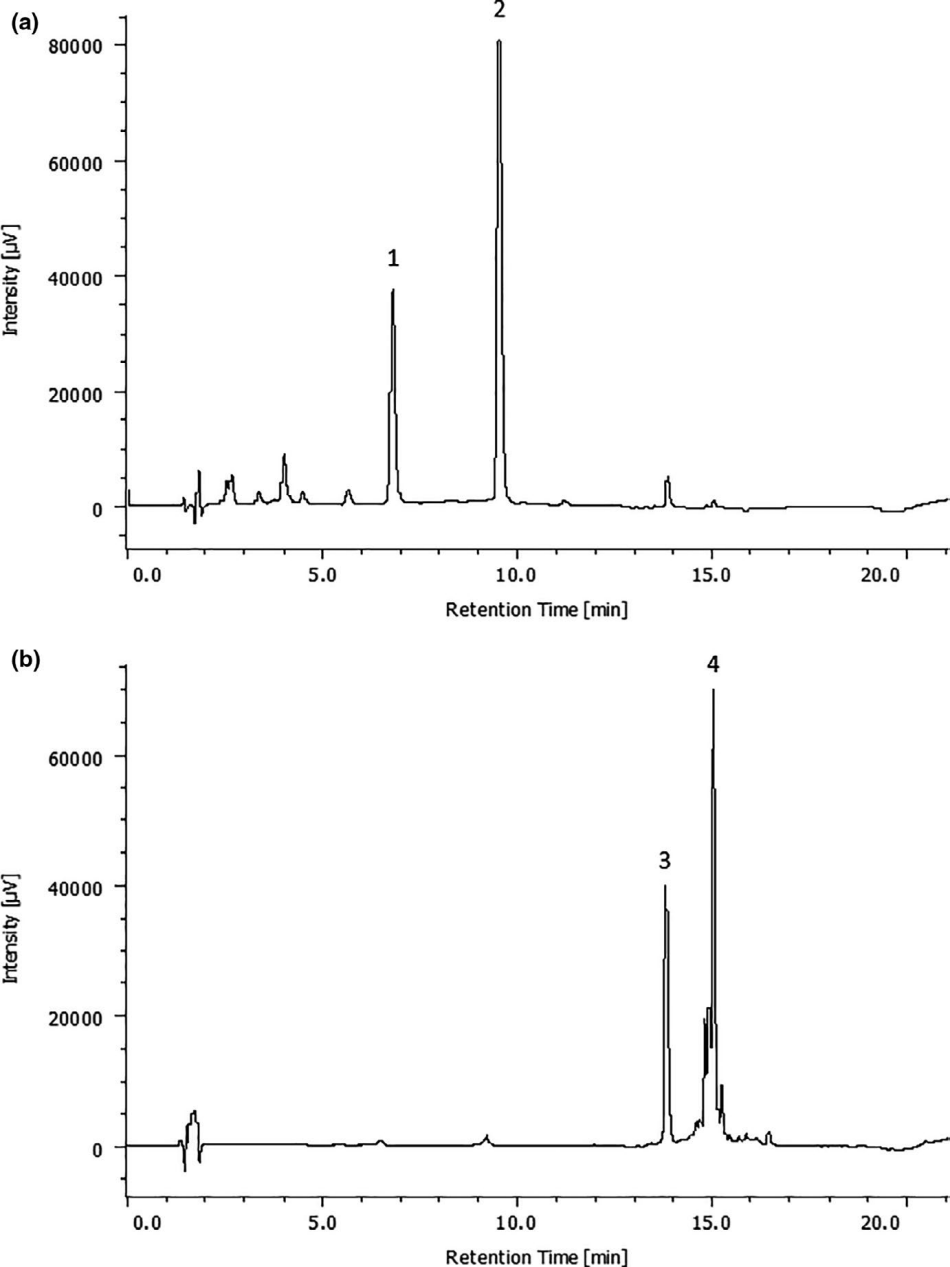
Representative chromatograms in 370 nm of the HPLC analysis for punicalagins and ellagic acid‐enriched extracts from pomegranate peel. (a) Peaks representing punicalagin (Pun)‐enriched extract (1 = *α* pun and 2 = *β* pun). (b) Peaks representing ellagic acid (EA)‐enriched extract (3 = ellagic acid‐hex and 4 = ellagic acid)

After the treatments with aqueous and ethanolic total extracts, we performed the separation of the main phenolic compounds (punicalagins and ellagic acid) to confirm whether each one had distinct effects or if they could act synergistically. HPLC chromatograms recorded at 370 nm shows the punicalagin‐enriched extract (peaks: 1 = *α* pun and 2 = *β* pun) and ellagic acid‐enriched extract (peaks: 3 = ellagic acid‐hex and 4 = ellagic acid) (Figure 6).

**FIGURE 7:**
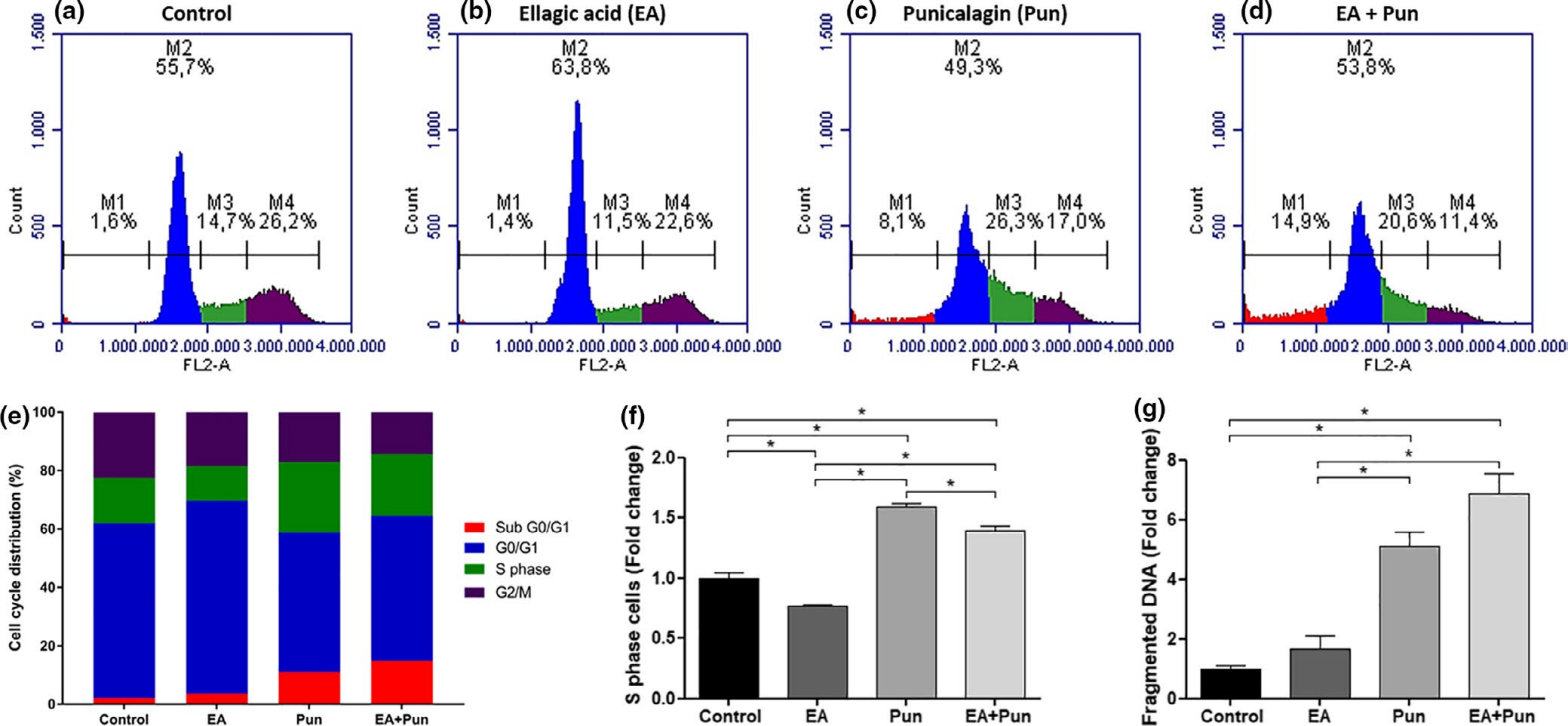
Cell cycle progression of THP‐1 cells treated with enriched fractions from pomegranate peel for 48 hr. (a) Control sample: THP‐1 cells were treated with 18 µl of ethanol (vehicle) (9 µl ethanol 30% + 9 µl ethanol 70%). (b) Treated sample: THP‐1 cells were treated with 18 µl of diluted ellagic acid extract (9 µl of extract in ethanol 70% + 9 µl of 30% ethanol). (c) Treated sample: THP‐1 cells were treated with 18 µl of a diluted punicalagin extract (9 µl of extract in ethanol 30% + 9 µl of 70% ethanol). (d) Treated sample: THP‐1 cells were treated with 18 µl of diluted ellagic acid and punicalagin extracts (9 µl of ellagic acid extract in ethanol 70% + 9 µl of punicalagin in ethanol 30%). In all samples, the cells were treated for 48 hr, and the cell cycle distribution was evaluated after propidium iodide (PI) staining. Data are representative of two independent experiments. (e) Analysis of the percentage of cells obtained of control and treated samples in sub G0/G1, G0/G1, S, and G2/M phases. (f and g) Analysis of the percentage of cells obtained in control and treated cells in the S phase (f) and with fragmented DNA (g). The results were presented in the fold change as mean ± *SEM* of two independent experiments. * indicates statistically significant differences (*p* < .05)

THP‐1 cells were treated with isolated compounds for 48 hr. Punicalagin‐enriched fraction (Pun) caused an S phase cell cycle arrest, raising the number of cells in this phase in about 58%, which was not increased by the addition of ellagic acid‐enriched fraction to the treatment (EA + Pun). However, EA + Pun treatment also presented a significant S phase impairment, rising about 39% the number of cells in this cell cycle phase. Besides, ellagic acid‐enriched fraction (EA) alone did not affect cell cycle progression (Figure 7).

We observed a significant increase in the percentage of cells with fragmented DNA in the treatment with punicalagin‐enriched extract (Pun) in about 5 times that was intensified to almost seven times by addition of ellagic acid‐enriched fraction to the treatment (EA + Pun) (Figure 7). However, the treatment with ellagic acid‐enriched fraction (EA) alone did not affect the percentage of cells with fragmented DNA (Figure 7).

**FIGURE 8:**
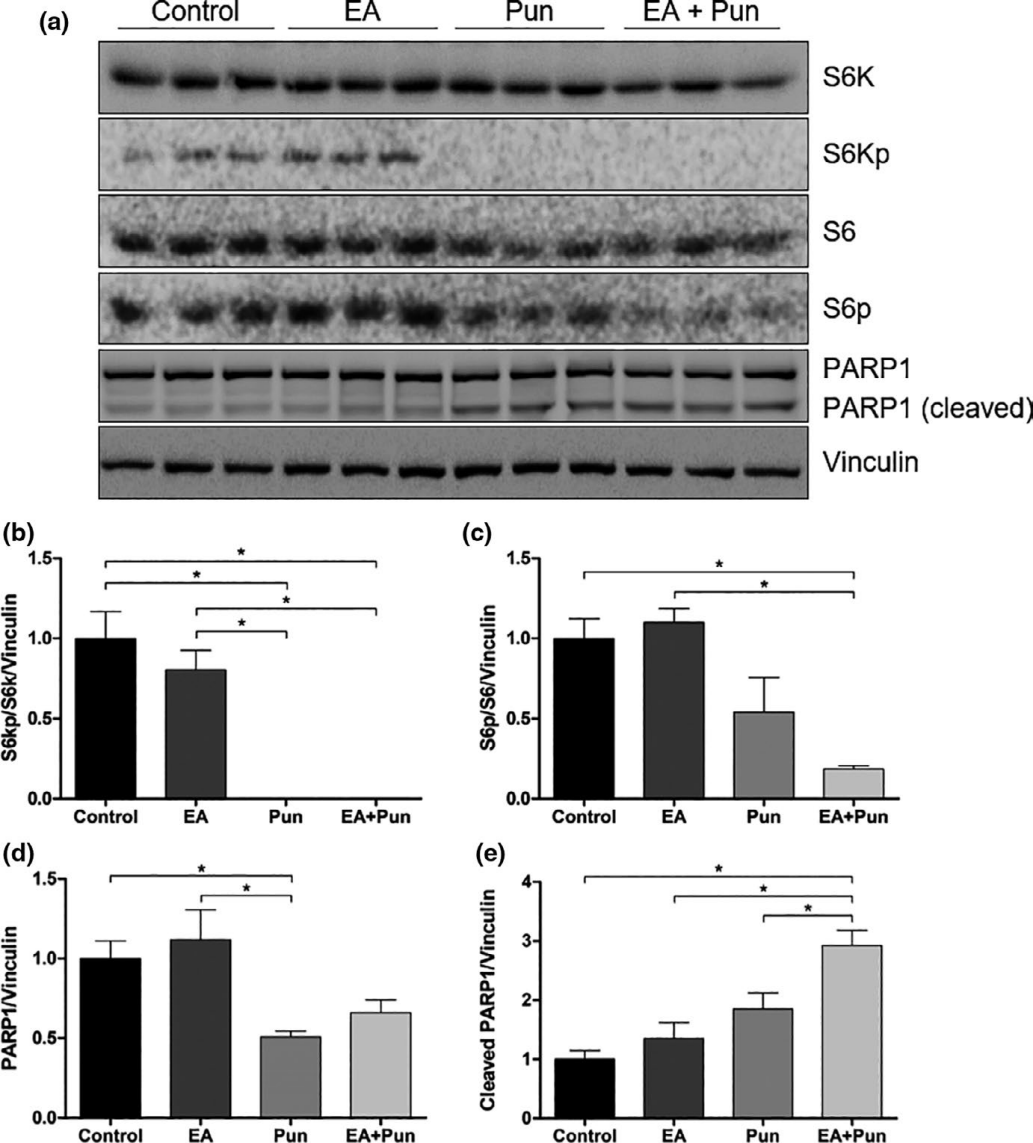
Effects on proteins expression in THP1 cells treated with enriched extracts from pomegranate peel for 48 hr. (a) Immunoblotting assay showing the expression of S6K, phosphorylated S6K (S6Kp), S6, phosphorylated S6 (S6p), PARP1, and cleaved PARP1 in THP1 cells after treatment with ethanol (control), ellagic acid (EA), punicalagin (Pun), and combination of ellagic acid and punicalagin (EA + Pun) for 48 hr. The phosphorylated and total forms were detected using specific antibodies, and anti‐vinculin antibody was used as the loading control. (b‐e) Graphic representation of the fold changes of (b) S6Kp/S6K (c) S6p/S6, (d) PARP1, and (e) cleaved PARP1. The ratios between phospho and total S6K; phospho and total S6; and PARP1 and cleaved PARP1 were normalized with vinculin. The results were presented in the fold change as mean ± *SEM* of two independent experiments. *indicates statistically significant differences (*p* < .05)

We also evaluated some proteins content involved with cell growth and apoptosis activation. The treatment with punicalagin‐enriched fraction (Pun) was able to significantly reduce the phosphorylation of S6K and S6 proteins, both downstream proteins of the growth‐related mTOR pathway (Figure 8). The treatment with ellagic acid‐enriched fraction (EA) only was not able to reduce those phosphorylations, whereas the treatment combining ellagic acid and punicalagin fractions (EA + Pun) had the same effects observed for punicalagin (Figure 8). Besides, it was observed a significant increase in the cleavage of PARP1 protein, indicating apoptosis activation in the treatment combining ellagic acid and punicalagin fractions (Figure 8).

## 4 DISCUSSION

One of the challenges in the field of phytochemistry is the use of technologies to improve the extraction yield and selectivity of several compounds from plants to maximize its functional properties. The conventional methods used to extract natural compounds include maceration, steam distillation, and Soxhlet extraction, whereas the nonconventional methods include ultrasound‐assisted extraction, microwave‐assisted extraction, supercritical fluid extraction, and pressurized liquid extraction (Chemat et al., 2017; Rostagno, & Prado, 2013). However, nonconventional extraction methods are becoming quite popular, especially the ultrasound‐assisted extraction, mainly due to its simplicity and inexpensiveness for application to industrial production on a large scale (Zhou et al., 2017). The cell walls can be broken by the cavitations provided by ultrasound, accelerating the release of the contents, and reducing the extraction time (Chemat et al., 2017). The use of ultrasound promises to improve the quality of some extracts. In addition to the extraction technique, several variables in the process can be adjusted to improve the extraction efficiency, such as temperature, solvent type, pressure, and ultrasound frequency (Carciochi, Manrique, & Dimitrov, 2015). The choice of the extraction solvent is critical, and it will determine the chemical profile of the extracts, such as the absolute and relative concentrations of the collected compounds (Rostagno, & Prado, 2013; Sumere et al., 2018).

In our study, we used ultrasound‐assisted extraction, and we tested a variety of solvents, including water and hydroalcoholic mixtures, to access the differences in the chemical profile of extracts obtained from pomegranate peel. Our results show that the most significant difference was on the concentration of the main phenolic compounds found in the peels (*α* pun, *β* pun, ellagic acid‐hex, and ellagic acid). We observed that the higher the solvent polarity, the greater the concentration of punicalagins in the extracts. Indeed, when we compare the amounts of *α* pun and *β* pun in the aqueous extract (562.26 ± 47.14 mg/L and 1,251.13 ± 22.21 mg/L, respectively) with the 70% ethanol extract (259.09 ± 19.71 mg/L and 506.55 ± 4.68 mg/L, respectively), we observe the highest concentration of punicalagins in the most polar solvent (water). Significant differences have been reported for punicalagin extraction from pomegranate peels using other types of solvents, such as methanol, ethanol, and ethyl acetate, which also affected the antioxidant activity of those extracts (Khalil et al., 2018). Methanolic extracts presented the highest concentration of punicalagins (110.00 ± 5.10 mg/g) and antioxidant activity (Khalil et al., 2018). Besides the solvent influence, another study observed differences in the punicalagin content and antioxidant activity of extracts obtained from pomegranate peels from three different varieties (Khalil, Khan, Shabbir, & Rahman, 2017). Similar to ours, both studies described punicalagins as the most predominant ellagitannin quantified on pomegranate peel extracts (Khalil et al., 2017, 2018). The solvents used in our study also affected the concentration of ellagic acid in the extracts. The highest concentration was observed in the 30% ethanol extract (93.01 mg/L), whereas the lowest in the aqueous extract (66.38 mg/L).

Regarding the use of the obtained extracts for the cell treatment, the solvent used in the extraction process could be toxic to the cells and present a significant effect on the cell cycle. To eliminate the solvent effects, the same solvents were used in the control samples. Leukemic monocyte THP‐1 cells were treated using the same volume of all extracts. The volume of treatment was defined based on the final ethanol concentration in the well, which could not exceed 1.25%. Ethanol toxicity tests in THP‐1 cells showed that this concentration was not able to affect the cell cycle progression and even the percentage of cells with fragmented DNA (data not shown). Based on that, the aqueous extract, in addition to presenting the highest content of punicalagins, would be used in higher volumes for the cell treatment, without causing any toxicity related to the solvent.

Pomegranate peel extracts have been shown to retard the proliferation of cells in several human cancer cell lines without causing toxicity (Panth et al., 2017; Settheetham & Ishida, 1995; Song et al., 2016). Tumor cells usually lose the ability to control the cellular division, passing through the cell cycle checkpoints and resulting in an uncontrolled proliferation status (Dash & El‐Deiry, 2004). Many compounds used for chemotherapy are based on the ability of these compounds to retard the cell cycle progression, through a cell cycle arrest, resulting in an impairment of the cell proliferation rate and, consequently, decrease the tumor growth (Zhivotovsky & Orrenius, 2010). Moreover, the cytotoxicity effects of the chemotherapeutic agents and the drug resistance in cancer are still a challenge (Housman et al., 2014). In this regard, many studies have been carried out to include natural compounds in therapeutic strategies (Khalid, Ayman, Rahman, Abdelkarim, & Najda, 2016; Safarzadeh, Sandoghchian Shotorbani, & Baradaran, 2014; Walczak, Marciniak, & Rajtar, 2017).

In this study, we showed that pomegranate peel aqueous extract was able to promote cell cycle impairment in the cell cycle phase that controls the DNA duplication (S phase) of THP‐1 cells. This effect corroborates to those previously reported for the treatment of leukemic cells with pomegranate juice (Dahlawi et al., 2012, 2013). Otherwise, another study observed a cell cycle arrest in a different cell cycle phase (G2/M) when using pomegranate peel extract obtained by another extraction method in a different cell line (K562 cells) (Asmaa et al., 2015). Besides that, the S phase is one of the targets of several anticancer drugs to reduce the proliferation of tumor cells, such as methotrexate, pralatrexate, 7‐Hydroxystaurosporine (UCN‐01), etoposide, and many others (Shapiro & Harper, 1999; Tazawa et al., 2014; Wood & Wu, 2015). However, these effects on cell cycle arrest were not observed when THP‐1 cells were treated with 70% ethanol extract, which suggests that this effect was due to the highest concentration of punicalagins in the aqueous extract.

Furthermore, we also observed that the extract produced using 30% ethanol as solvent presents the highest amount of ellagic acid and total phenolic compounds. The treatment of THP‐1 cells using this extract showed the best responses to raise the number of cells with fragmented DNA, which was almost seven times higher than the untreated cells. Similar to ours, a study performed with two human Burkitt's lymphoma cell lines, Raji and P3HR‐1, showed that aqueous pomegranate peel extract resulted in apoptotic DNA fragmentation and suppression of growth (Settheetham & Ishida, 1995). The increase of cells with fragmented DNA indicates apoptosis induction, an early response cell death, and it is a useful marker for predicting tumor response to anticancer treatment (Fulda, 2009). Our data revealed that the increase in the percentage of cells with fragmented DNA might be related to the high concentration of ellagic acid and total phenolic compounds in this extract.

We obtained punicalagin and ellagic acid‐enriched fractions from pomegranate peel extracts. The enriched extracts were also tested against THP‐1 cells. We confirmed that punicalagins are the main responsible for the observed effects in the S phase cell cycle arrest. We also observed that ellagic acid acts synergistically with punicalagin, but not alone, in the apoptosis induction. Our data are in agreement with another one using pomegranate juice for cell treatment (Dahlawi et al., 2013). After the treatment with the enriched fractions, we also evaluated some protein contents related to cell growth and apoptosis. Increased levels of cleaved PARP1 in the treatment combining ellagic acid and punicalagin fractions confirmed the apoptosis induction by the treatment. PARP1 protein responds do DNA damage and its cleavage is a known marker of apoptosis induction (Burkle & Virag, 2013; Diamantopoulos et al., 2014; Pinton et al., 2013). Besides, the mTOR/S6K signaling pathway was preferentially inhibited by the punicalagin‐enriched fraction, which has also been observed by other studies (Adhami, Siddiqui, Syed, Lall, & Mukhtar, 2012; Banerjee, Kim, Talcott, & Mertens‐Talcott, 2013; Kim et al., 2016; Wang, Chen, Longtine, & Nelson, 2016). The mTOR/S6K signaling pathway is an important regulator of cell growth and it is related to tumorigenesis and cancer aggressiveness (Amaral et al., 2016; Menon & Manning, 2008; Tam et al., 2009; Tavares et al., 2015).

Our findings show the importance of studying the nonedible parts of pomegranate and other fruits, which are usually considered byproducts of the food industry. The use of nonconventional methods of extraction could also improve the quality of the extracts, and the ideal solvent needs to be used to obtain an extract with specific desired compounds. Since punicalagins and its metabolites are the most studied phenolic compounds from pomegranate peel, it seems promising to advance the chemical and functional characterization of the whole extract to validate its use as therapeutics. Our findings reveal that the solvent choice is determinant for the chemical profile and the observed effects against cancer cells.

## 5 CONCLUSION

In conclusion, our results showed that the composition and concentration of phenolic compounds obtained were significantly influenced by the use of different solvents as well as their biological activities. The highest concentration of punicalagins in the aqueous extract was the main responsible for the S phase cell cycle arrest induced by this treatment. On the other hand, the increase in the percentage of cells with fragmented DNA, observed in the treatment using 30% ethanol extract, was due to the highest concentration of ellagic acid and total phenolic compounds. Both results were confirmed by the treatment with punicalagins and ellagic acid‐enriched fractions from pomegranate peel. As demonstrated by this study, *in vitro* experiments can be a valuable screening tool for future animal and human studies for the use of pomegranate peel extracts as therapeutics in the treatment and prevention of malignancies.

## CONFLICT OF INTERESTS

We wish to confirm that there are no conflicts of interest, financial, or otherwise associated with this publication.

## AUTHORS’ CONTRIBUTIONS

ADL and MAR conceived and supervised the study; LT, BRS, FMS, MAR, and ADL designed the experiments; LT, BRS, MCS, NFP, ACA, MNE, and MAR performed the experiments; and LT, BRS, MCS, NFP, FMS, MAR, and ADL analyzed the data, interpreted the results, and wrote the manuscript.
